# *Helicobacter pylori* infection induced gastric cancer; advance in gastric stem cell research and the remaining challenges

**DOI:** 10.1186/1757-4749-4-18

**Published:** 2012-12-08

**Authors:** Song-Ze Ding, Peng-Yuan Zheng

**Affiliations:** 1 Department of Gastroenterology, Henan Provincial People’s Hospital, Zhengzhou University, Zhengzhou, Henan, 450000, China; 2 Department of Toxicology, University of Kentucky, Lexington, KY, 40503, USA; 3 Department of Gastroenterology, The Second Affiliated Hospital, Zhengzhou University, Zhengzhou, Henan, 450014, China

**Keywords:** *Helicobacter pylori (H. pylori)*, Cancer, Stem cell, Gastric epithelial cells, Epigenetics

## Abstract

*Helicobacter pylori* infection is the major cause of gastric cancer, which remains an important health care challenge. Recent investigation in gastric stem cell or progenitor cell biology has uncovered valuable information in understanding the gastric gland renewal and maintenance of homeostasis, they also provide clues for further defining the mechanisms by which gastric cancer may originate and progress. Lgr5, Villin-promoter, TFF2-mRNA and Mist have recently been identified as gastric stem/progenitor cell markers; their identification enriched our understanding on the gastric stem cell pathobiology during chronic inflammation and metaplasia. In addition, advance in gastric cancer stem cell markers such as CD44, CD90, CD133, Musashi-1 reveal novel information on tumor cell behavior and disease progression implicated for therapeutics. However, two critical questions remain to be of considerable challenges for future exploration; one is how *H. pylori* or chronic inflammation affects gastric stem cell or their progenitors, which give rise to mucus-, acid-, pepsinogen-, and hormone-secreting cell lineages. Another one is how bacterial infection or inflammation induces oncogenic transformation and propagates into tumors. Focus on the interactions of *H. pylori* with gastric stem/progenitor cells and their microenvironment will be instrumental to decipher the initiation and origin of gastric cancer. Future studies in these areas will be critical to uncover molecular mechanisms of chronic inflammation-mediated oncogenic transformation and provide options for cancer prevention and intervention. We review recent progress and discuss future research directions in these important research fields.

## Introduction

*Helicobacter pylori*, a microaerophilic, spiral-shaped Gram-negative bacterium, colonized in human stomach, is the major cause of chronic gastritis, peptic ulcers, and gastric malignancies, including gastric non-cardia adenocarcinoma and mucosal-associated lymphoid tissue (MALT) lymphoma [1]. Epidemiologically, *H. pylori* infects half of the world’s population, although most infected persons have no clinical presentation, approximately 1% infected people will develop into gastric cancer [2]. Despite ample amount of efforts in investigating its pathogenesis and host-pathogen interactions, the molecular mechanisms of how *H. pylori* infection induces gastric cancer remain poorly understood.

Gastric cancer (GC) is the second leading cause of cancer related death among all cancers, next only to lung cancer. Although the relationship between *H. pylori* infection and gastric cancer has been very well established, the mechanisms of how tumor initiation and the early development remain elusive. Studies in past decades have suggested that stem cells play a pivotal role in the initiation of gastric cancers, and the tumor can originate from bone marrow derived cells [3]. However, it is not clear how they give rise to tumors after inflammatory insult or bacterial infection; in this work, we briefly discuss the current progress and important challenges for future research.

### *H. pylori* virulence factors and host cell signaling in gastric epithelial cells

Four major virulence factors have been identified from *H. pylori* (Table 1)*,* including cytotoxin-associated antigen A (CagA), *cag-*pathogenicity island (*cag*PAI), vacuolating cytotoxin (VacA), and outer membrane proteins (OMPs). *H. pylori cag*PAI is a 40 kilobase region of *H. pylori* genome that encodes about 30 genes, some of them encode type four secretion system (TFSS), which are essential for pathogenesis and are responsible for delivery of CagA protein and peptidoglycan (PGN) into host cells [4,5]. *H. pylori* CagA is encoded by *cagA* gene within the *cag*PAI, has a molecular weight around 120–145 kDa, induces multiple host cell singaling [6]. VacA is an 88-kDa bacterial protein and toxin, which can undergo limited proteolytic cleavage to yield two p33 and p55 subunits. Major effects of VacA include causing host cell vacuolization, apoptosis and inhibiting cell proliferation [7]. OMPs including Hop (Helicobacter outer membrane porins) and Hor (Hop-related proteins) groups proteins that include 33 members, their functions are associated with enhanced gastric mucosa inflammation and are necessary for bacteria adhesion to epithelial cells [8]. Infection with *cag*PAI- and *cagA-*positive *H. pylori* strains is linked to increased gastric cancer risk [1,2].

**Table 1:** **Major *H. pylori *virulence factors and their functions**

**Name**	**Size/genes**	**Effects in host cell**	**Reference**
CagA	120-140 kDa	Oncoprotein, disrupt cell polarity, multiple cell signaling	[6]
*cag*PAI	about 30 genes	Type VI secretion system for CagA, PGN injection, and NF-κB activation	[4,5]
VacA	88 kDa protein	Vacuolization, apoptosis and inhibiting cell proliferation	[7]
OMPs	33 members	Inflammation and adhesion	[8]

*CagA* cytotoxin-associated antigen A; *cagPAI cag*-pathogenicity island; *VacA* vacuolating cytotoxin; *PGN* peptidoglycan; *OMPs* outer membrane proteins.

*H. pylori* CagA and PGN are injected into host cell cytoplasm following its adherence to gastric epithelial cells, and induce CagA- and *cag*PAI-dependent effects on epithelial cells [4,5]. The basis for pathogenesis and clinical presentation is that *H. pylori* virulence factors activate multiple intracellular pathways in epithelial cells, such as mitogen-activated protein kinases (MAPK), NF-κB, activator protein (AP)-1, Wnt/β-catenin, signal transducer and activator of transcription 3 (STAT3) and phosphatidylinositol 3-kinase (PI3K). Their expression lead to increased inflammatory cytokine production, immune cell infiltration, affecting host cell apoptosis, proliferation and differentiation, finally result in clinical presentation and epithelial cell oncogenic transformation [2,9].

CagA specific effects include activation of Src-homology 2 domain-containing tyrosine phosphatase (SHP-2), upstream of Ras/Raf/Mek/Erk cascade; disruption of epithelial cell barrier function through dephosphorylation of cortactin, ezrin, associate with ZO-1; and binding to Par1/MarkII complex that damage normal cell contact, cell polarity, and epithelial cell permeability [6]. CagA also activates nuclear factor of activated T-cells (NFAT) signal pathway, and interacts with E-cadherin to deregulate β-catenin signaling, which induces β-catenin downstream genes expression such as *CDX1* and promotes cell intestinal transdifferentiation [10].

Major *cag*PAI-dependent host signaling involve introducing PGN into host cell cytoplasm and activate NF-κB by Nod 1 signaling [4,5]. Activation of NF-κB mediates the expression of multiple genes involved in *H. pylori*-induced host responses, such as expression of interleukin (IL)-6, -8. In addition, recent studies indicate *H. pylori* infection also causing epigenetic changes in gastric epithelial cells including DNA methylation and histone modifications [9]. Further investigation in these areas will lead to the discovery of important molecular mechanisms in bacteria-induced gastric cancer.

## Bacterial and host factors involved in gastric cancer related to *H. pylori* infection

Infection with *H. pylori* in mice and gerbils, and infection of *H. felis* in mice results in stomach inflammation and eventually leads to the step-wise histopathologic changes presented as chronic atrophic gastritis, intestinal metaplasia, dysplasia, and gastric adenocarcinoma [11-13]. The results implicate synergistic interactions among *Helicobacter* components, inflammation and host factors for gastric carcinogenesis. Recent studies have begun to address individual bacterial and host components in pathogenesis.

### *H. pylori* CagA

*H. pylori* CagA is a bacterial oncoprotein and is sufficient to generate gastric cancer alone in mice. Transgenic mice that overexpress bacterial CagA protein alone induce multiple malignancies [14], including gastric epithelial hyperplasia, hyperplastic polyps, gastrointestinal carcinomas, and hematological malignancies. The mice have no signs of gastritis or systemic inflammation, and the cancers are cell autonomous. These results directly indicate the role of CagA in gastric tumorigenesis, although the detailed mechanisms remain to be explored further.

### Wnt/Î²-catenin and cyclooxygenase 2 pathways

*H. pylori* infection activates Wnt/β-catenin and cyclooxygenase 2 (COX2)/prostaglandin E(2) pathways that play a critical role in gastric carcinogenesis. It has been known that *H. pylori* infection induces β-catenin and p120 expression which mediate peroxisome proliferator-activated receptor δ expression in gastric epithelial cells, and promotes gastric epithelial cell proliferation, these effects have been attributed to the *cag* secretion system substrates CagA and peptidoglycan [15]. Activation of β-catenin pathway also leads to targeted transcriptional up-regulation of genes that implicated in carcinogenesis, such as NFAT signaling [10,11].

Transgenic mice that overexpress COX2 and Wnt1 each alone do not produce tumor, but cross the two components to generate K19-Wnt1/C2mE mice, which express both COX2 and Wnt1, induce cancer in these mice. In the stomach of these mice, macrophages are also recruited to the gastric mucosa, and promote tumor formation [13,16], suggesting combined actions or synergistic effects of COX2 and Wnt signaling pathways on the cancer formation processes.

### Inflammatory cytokines

Infection of *H. pylori* also disrupts gastric homeostasis and induces multiple inflammatory cytokine production within local mucosal, components such as IL-1β, TNF-α, and IL-10 genotypes are associated with increased risk for developing gastric cancer [17-19].

IL-1β is a proinflammatory cytokine involved in inflammation and immunity. IL-1β polymorphisms are associated with enhanced IL-1β production and increased risk of gastric cancer [19], IL-1β also inhibits gastric acid secretion. In transgenic mice, stomach specific overexpression IL-1β induces stepwise spontaneous gastric inflammation, metaplasia, dysplasia, and carcinoma. *H. felis* infection results in more rapid progression to gastric atrophy and cancer. Overexpression of IL-1β also mobilizes myeloid-derived suppressor cells and induces NF-κB activation as well as its downstream genes IL-6, TNF-α expression in these cells. In addition, IL-1β alone is sufficient to induce gastric preneoplasia [17]; however, it is not clear the mechanisms of how IL-1β over expression itself finally results in oncogenic transformation at present.

Interestingly, other inflammatory mediators can exert opposite effects. One example is IFN-γ, which is produced primarily by activated T cells, natural killer cells and is a key mediator of innate and adaptive immunity. IFN-γ mediates responses to bacterial infection and autoimmune disease, and acts as tumor suppressor [18]. In mice, stomach specific overexpression of IFN-γ alone has minimal effects on gastric mucosa, but inhibits IL-1β- and *H. felis*–induced gastritis and neoplasia. The mechanism has been attributed to IFN-γ inhibits gastric epithelial cell proliferation, accelerates apoptosis of gastric T lymphocytes and decreases production of proinflammatory Th1 and Th17 cytokines. These effects may balance epithelial cell proliferation, restrain inflammation, and ultimately inhibit tumor formation [18]. Therefore, disruption of host cell inflammatory cytokine production participates in gastric oncogenesis.

### Trefoil factors

The trefoil factor family proteins (TFF1, 2, 3), regulate mucosal repair and suppress tumor formation in stomach. TFF1 and TFF2 have recently been identified to play a critical role in antagonizing gastric carcinogenesis [20,21]. *Tff1* acts as a tumor suppressor gene and its deficiency results in spontaneous gastric cancer in mice [21]. *Tff2* deficient mice only display subtle alterations in mucosal proliferation; parietal cell activation presented as increased acid secretion and increased susceptibility to NSAID injury. Genetic ablation of *Tff2* accelerates tumor growth and promotes preneoplastic lesions in mice antrum [20]. In these mice, imbalance of hormone and cytokine production was noted, with decreased gastrokine 1, 2 mRNA production and increased Th1 response, decreased Th2 response (IL-1α, β and interferon-γ, reduced IL-13, IL-4) [20,21].

Both TFF1 and TFF2 expression are frequently lost in gastric cancer and aberrant promoter methylation has been suggested to be an important mechanism. Interestingly, *H. felis* infection enhances the loss of TFF1, and *H. pylori* (SS1 strain) infection increases *Tff2* promoter methylation in mice which results in the lower level of protein expression [20,21]. Therefore, these data reveal protective effects of TFF proteins and their reduction promote gastric carcinogenesis.

Together, the above systems have clearly advanced our understanding of the role of *H. pylori* or host factors in gastric carcinogenesis; although the data are fairly fragmentary, they can be converged and potentially impact on gastric stem or progenitor cells toward carcinogenesis. In fact, the role of stem or progenitor cells in these model systems has just begun to be appreciated. Studies have now moved to address the tumor initiation and malignant transformation by focusing on stem cell, microenvironment, and recruitment of various progenitor cells that contribute to tumor growth, which will be discussed in more detail below.

## Gastric stem or progenitor cells and their markers

Gastric epithelial cells that make up the gastric gland in mucosa are pit, parietal, neck, and zymogenic cells, as well as the progenitor cells. Two important limitations in stem cell research are lack of *in vitro* culture system and shortage of specific markers for different stages of stem cells. Recent studies begin to identify these cells by mouse genetics lineage tracing analysis, which is a powerful tool for characterization and validation of stem cell markers and their functions.

The original concept of gastric cancer stem cell hypothesis is that cells located in the isthmus region of gastric gland, the morphologically immature cell with less organoid, are multipotent stem cells; they are responsible for generating the four major cell types of gastric gland [22]. These stem cells give rise to transit amplifying (daughter/progenitor) cells, which can differentiate into all mature cell types. During this process, descendants derived from stem cells undergo a complex bipolar migration from neck/isthmus region, moving either upward or downward. In mouse stomach, all three types of progenitor cells (prepit, preneck, and preparietal cells) have been found to originate from multipotent granule-free cells in isthmus region [22]. However, this model has recently been enriched with the discovery of proliferating Villin-promoter- and Lgr5-marked cells (see below) at the base and various locations of pyloric glands, which exhibit both self-renewal and the ability to generate all differentiated epithelial cell types of the pyloric gland [23,24]. These observations have opened new avenues to investigate gastric stem cell biology and pathobiology.

### Genetic linage tracing marked gastric stem cells

Several recently identified gastric stem or progenitor cell markers using genetic linage tracing technology are listed in Table 2.

**Table 2:** Gastric stem and progenitor cell markers

**Name**	**Location**	**Function**	**Reference**
Lgr5	Antral, gland base	Give rise to gastric unit, all four types of cells	[23]
Villin-promoter	Antral, gland base	Give rise to gastric unit, all four types of cells	[24]
Tff2 mRNA	Corpus, gland base	Give rise to only parietal, neck, and chief cells	[27]
Mist1 (BHLHA15)	Corpus, gland base	Chief cell for SPEM generation	[28]

*Lgr5* leucine-rich repeat-containing G protein-coupled receptor5 (Lgr5); *Villin-promoter* Villin-promoter marked stem cells; *Tff2 mRNA*, Trifold factor 2 mRNA marked stem cells; *Mist1 (BHLHA15)*, basic helix-loop-helix family, member a15.

#### Lgr5 marked gastric stem cells

Using *in vivo* linage tracing analysis, Barker and colleagues [23] demonstrate that Lgr5 (leucine-rich repeat-containing G protein-coupled receptor 5, Gpr49) marked cells are long live; reside in the gland base, morphologically immature, with a large nuclear-to-cytoplasmic ratio and limited organelles. Lgr5 is a Wnt target gene identified in colon cancer cell lines, and intestinal crypts. It is recently identified as a novel stem cell marker of stomach, intestinal epithelium and hair follicle [23].

In neonatal mice stomach [23], Lgr5 is expressed at the base of corpus and pyloric glands, whereas in adult mice Lgr5 is predominantly restricted to the base of mature pyloric glands. In contrast to the common concept that stem cell are quiescent, Lgr5 marked cells, proliferate fast and are able to build up the entire gastric gland over a short time. During *in vitro* culture, single Lgr5 positive cell efficiently generated long-lived organoids resembling mature pyloric epithelium with self-renewing capability, architecture and cell composition.

Study the transcriptome of Lgr5 cells [23] reveal total of 153 changed genes expression in Lgr5 stem cell/daughter cell population, many are Wnt target genes, including CD44, Sox9, Sord, Prss23, Sp5, indicating canonical Wnt signaling activity at the base of pyloric glands. Several enteroendocrine-specific genes, including Chromogranin A&B, Somatostatin, and Gastrin G, are highly upregulated in Lgr5 daughter cell population, implying rapid differentiation toward the enteroendocrine lineage.

Lgr5 stem cells are also tumorigenic, mice with deletion of APC gene, an essential Wnt signaling gene resulted β-catenin upregulation at the base of pyloric glands, and within 2–3 weeks, these Lgr5 cells can grow into highly proliferative, β-catenin-positive adenomas. This effect is not detected in gastric corpus, confirming the absence of Lgr5 in this region [23].

Because of these exciting discoveries, distribution of Lgr5 cells is further examined in human stomach. Immunostaining revealed relocation of Lgr5 cells in different stages of gastric diseases [25]. In non-neoplastic stomach mucosa, Lgr5 cells are found predominantly in mucous neck region; during intestinal metaplasia, they localize at the crypt base; and in GC, Lgr5 cells are present at the luminal surface, tumour center and the invasion front. Distribution of Lgr5 cells in tumor center and invasion front of GC correlates well with tumor growth and nodal spread. In addition, patients with Lgr5 positive GCs have a shorter median survival than patients with Lgr5 negative GCs. Tumor tissues from esophagus, stomach, liver, pancreas, colon and rectum all show significantly more Lgr5 cells and higher levels of Lgr5-mRNA expression when compared with non-tumor tissues [25].

Together these observations in mice and human have documented that stem cells are far more mobile and less restricted to the positions as they were originally thoughts. In addition, Lgr5 marked cancer cells can proliferate towards various directions inside the mucosa, indicating an invasive growth property and imply that they are crucial for gastric cancer development and progression. Further studies are required to understand their roles in gastric tumorigenesis especially during the chronic *H. pylori* infection setting. Interestingly, the trend has already begun; pilot report has shown that *H. pylori* infection is associated with increased DNA damage of Lgr5 positive cells in gastric cancer patients [26].

#### Villin-promoter marked gastric stem cells

Villin is an actin-binding-protein, mainly expressed in the brush border of intestinal epithelium, plays a key role in the morphogenesis of microvilli to form membrane protrusions that increase cell surface area and involve in cell absorption, secretion and adhesion. Villin is highly expressed in intestinal cell and largely devoid from gastric epithelial cell.

Lineage tracing studies confirm that Villin-promoter marked cells can be found in mice gastric epithelium, they are quiescent and locate at, or below isthmus in the bottom third part of pyloric glands and rare in corpus [24]. These cells give rise to multiple gastric lineages of antral glands, including surface pit cell (mucous gland cells), enteroendocrine cells, and parietal cells. However, endogenous Villin protein expression is not observed in the gastric epithelia of these mice probably due to gene structural disruptions; therefore it is difficult to establish the precise relationship of active and quiescent stem cell populations. Similar to Lgr5, Villin-promoter marked stem cell population are also absent in the corpus region and indicate different stem cell biology in this region of stomach [24].

One important finding from this elegant work is that forced IFN-γ exposure in mice to mimic inflammation cause remarkable expansion of Villin-promoter marked cell population in antral mucosa [24]. The marked cells can be found to locate at various positions in the lower portion of the gland, between isthmus and gland tip, and situated on the opposite side of gland base, suggesting inflammation regulates these cell proliferation and migration. While using CDX2 transgenic mice, a model to induce intestinal metaplasia, find no significant change in both cell number and locations, implicating Villin-promoter marked cells do not give rise to the metaplasia. It would be interesting to see if other inflammatory mediators might impact on these cells and alter their amplification and mobility as well.

#### Trefoil factor 2 mRNA marked gastric stem/progenitor cells

In gastric gland, mucus neck cells locate below the isthmus region and express trefoil factor family 2 proteins; but TFF2 mRNA transcripts are concentrated in cells above the neck region in normal corpus mucosa. This positional change suggests that TFF2 mRNA transcript-expressing cells (TTE) might be gastric progenitor cells and TFF2 mRNA transcripts may be a marker [27].

In oxyntic mucosa [27], lineage tracing demonstrate that TFF2 mRNA marked cells migrate toward the bottom of the gland, give rise to parietal, mucous neck, and chief (zymogenic) cell lineages, but not enterochromaffin-like-cell. Surface mucus cells are not derived from TTE cells and the progeny of the TTE lineage do not survive beyond 200 days [27]. In contrast to corpus, in antrum, TTE cells do not appear to migrate, nor are they represent any type of stem or progenitor cell in distal stomach, suggesting TTE marked cells only located in the gastric corpus. However, functional data are not available from this study, future studies are required to understand its role in gastric gland renewal and differentiation during inflammation.

#### *Mist1* marked gastric stem/progenitor cells

Mist1 (BHLHA15) is a transcription factor, expressed in chief cells and is critical for chief cell basal localization and maintenance. It is expressed at the base of gastric gland and in cells located at transition zones between the neck and base region with partial neck-chief cell characteristics [28].

Lineage tracing in acute and chronic oxyntic atrophy mice models with Mist1 expressing cells (using chemical L-635 treatment and *H. felis* infection) demonstrate that chief cell can give rise to entire spasmolytic polypeptide-expressing metaplasia (SPEM) lineage. SPEM is a recently appreciated form of gastric mucosal metaplasia in addition to intestinal metaplasia, SPEM get it name due to it expresses TFF2 (spasmolytic polypeptide), also called pseudopyloric metaplasia or mucous metaplasia or antralization of the corpus [29]. Chief cells may therefore represent progenitor cells for metaplasia [28]. Loss of parietal cells induces transdifferentiation of chief cell into SPEM and this metaplasia can undergo expansion under the influence of acute or chronic inflammation.

Gastric atrophy of corpus and body are associated with the development of SPEM, and SPEM is strongly associated with the development of gastric cancer similar to intestinal metaplasia. Both SPEM and intestinal metaplasia are observed in the stomachs of gastric cancer patients. SPEM possesses the characteristics of antral metaplasia and express TFF2 and MUC6, while intestinal metaplasia demonstrates clear lineage characteristics of duodenum of intestines, with expression of TFF3 and MUC2 [28,29]. At present, it is not clear if gastric cancer might arise from either one or none of both types of metaplasia, future work are warranted to evaluate their relationship and the role of SPEM in gastric carcinogenesis.

### Putative gastric stem cell and cancer stem cell markers

For brief summary, a few recently reported putative gastric stem cell and cancer stem cell markers are listed in Table 3.

**Table 3:** Putative gastric stem cell and cancer stem cell markers

**Name**	**Location**	**Self-renewal**	**Xenograft**	**Reference**
CD44	gland base	sphere formation	tumorigenic	[32]
CD90	n/t	sphere formation	tumorigenic	[35]
CD133	gland base	yes	tumorigenic	[38]
Musashi-1	antral, isthmus/neck	n/t	n/t	[41]
pSmad2/3L-Thr	isthmus region	n/t	n/t	[43]

*CD* Cluster of differentiation; *n/t* not tested; *pSmad2/3L-Thr* Smad2/3 phosphorylated at specific linker threonine.

#### Cluster of differentiation 44 (CD44)

CD44 is a cell surface transmembrane glycoprotein that acts as a receptor for extracellular matrices such as hyaluronic acid, its function involves in cell–cell interaction, cell adhesion and migration. CD44 is a known downstream target of Wnt/β-catenin pathway and is expressed in many types of tissues, and solid tumors such as gastric, pancreas, colon cancers [30-33]. CD44 has a variety of splicing variants (CD44v) [30] and is identified as a solid tumor stem cell marker in breast cancer [31]. CD44 expressing cells have cancer stem cell (CSC) features including; tumorigenic, able to self-renewal and generate phenotypically diverse (mixed populations of) nontumorigenic cells.

In SCID (severe combined immunodeficiency) mice, CD44 positive gastric cancer cells are able to induce tumor [32], knockdown CD44 by short hairpin RNA decreases the tumorigenicity. Furthermore, these cells are resistant to chemotherapy or radiotherapy. In the same experiment, other potential CSC markers, such as CD24, CD133, CD166, stage-specific embryonic antigen-1 (SSEA-1), and SSEA-4, or sorting for side population of cells do not show any correlation with tumorigenicity *in vitro* or *in vivo*[32]. In addition, recent reports indicate CD44, ADAM17 and another putative stem cell marker, Musashi-1 coexpresses with stem cell markers Lgr5 in both normal and gastric cancer patient tissues [25].

Expression of CD44 variants in gastric cancer patients such as CD44v6 and CD44v9 are associated with progression of gastric cancer [30]. Expression of CD44v6 is also associated with lymph node metastasis, invasion and pathological grade of tumor with advanced stages, patients with CD44v6 positive tumors have a lower survival rate [34]. Cd44v9 is not expressed on gastric epithelium of *H. pylori* negative individual, but is present during *H. pylori* infection [30]. However, whether CD44 variants can serve as cancer stem cell makers is waiting for further investigations.

#### Cluster of differentiation 90 (CD90)

CD90 (Thy-1) is a cell surface glycoprotein, molecular weight at 25–37 kDa. In mice, it is expressed in thymocytes, neuron, T cells; in human, CD90 is expressed in endothelial, smooth muscle, fibroblasts and a variety of stem cells; including bone marrow derived mesenchymal stem cells, hepatic stem/progenitor cells, hematopoietic stem cells and keratinocytic stem cells [35,36]. CD90 marked cancer stem cells have been identified from human liver cancer, murine breast tumor and in clinical gastrointestinal stromal tumor samples [36,37].

CD90 is recently reported to characterize CSC population in gastric primary tumors [35]. In isolated human primary gastric tumor cells, CD90 acts as a surface marker and can be enriched under non-adherent, serum-free and sphere-forming conditions. CD90 positive cells separated from tumor tissue possess a higher ability to initiate tumor *in vivo* and reestablish the cellular hierarchy of tumors from single-cell implantation in nude mice model, higher proportion of CD90 positive cells correlate with higher *in vivo* tumorigenicity in mice [35].

Gene expression analysis indicates stem cell-related genes are upregulated in sphere-cultured cells, including CD90, Oct4, Sox2, Notch1 and ALDH1. Oct4 and Sox2 are known embryonic stem cell factors essential for pluripotency and self-renewal of embryonic stem cells. Notch1 and ALDH1 are markers in both normal and malignant stem and progenitor cells, but CD133 gene expression is not increased in these cells [35]. In addition, blocking ERBB2 pathway can suppress tumorigenicity, and reduce tumor mass/growth when combined with traditional chemotherapy [35]. These results suggest CD90 can be a potential cancer stem cell marker and implicated for monitoring gastric cancers treatment.

#### CD133/Prominin 1 (Prom1)

CD133 is a cell surface membrane protein and a universal marker for tissue stem/progenitor cells, as well as CSC; its mouse homologue is Prominin 1. CD133 has been under intensive investigation as a cancer stem cell marker in various solid tumors, including gastric, liver, colorectal, lung, ovarian, prostate, and breast cancers [33,38].

In mice, Prom1 protein expression is detected at the apical membranes of Lgr5 positive intestinal stem cells located at the base of crypts, coexpresses with Lgr5, but is also expressed on transit-amplifying progenitors. Linage tracing analyses demonstrate that Prom1 marks both Lgr5 intestinal stem cells and transit-amplifying progenitor cells, generates the entire intestinal epithelium, and is therefore the small intestinal stem cell marker [39]. In human stomach, CD133 does not coexpress with stem cell markers Lgr5 [25]. In gastric cancer patients, CD133 is expressed in more than 50% of the cases, and its expression appears as an independent prognostic marker for poor patient prognosis, high CD133 is associated with poor patient survival rates [40].

Not every gastric epithelial cell line expresses CD133, probably due to their differentiation stage varies [38]. In gastric tumor cells lines, CD133(+) cells can form both CD133(+) and CD133(−) cells; but CD133(−) cells form only CD133(−) cells, indicating CD133(+) cells retain the characteristics of self-renewal. When injected into nude mice, both cells form tumors, but with different differentiation stages; CD133(−) cell lines form poorly differentiated tumor while CD133(+) cell lines form well differentiated tumor [38]. However, using freshly isolated primary gastric cancer cells, CD133 expression is not correlated with tumorigenesis both *in vitro* and *in vivo*[33]. Investigation is now focusing on clinically targeting CD133 and its application in patient outcome prediction and cancer therapy.

#### Musashi-1

Musashi-1 (Msi-1), a RNA binding protein associated with asymmetric divisions in neural progenitor cells, is identified as a putative stem cell and early linage marker in mouse intestine [41]. In human stomach, immunostaining show that Msi-1 positive cells are located at isthmus/neck region of adult antrum, mixed with proliferating cell nuclear antigen (PCNA) positive cells; but does not colocalize with PCNA or Ki67, which are markers for cell proliferation. Msi-1 colocalizes partially with MUC5AC and MUC6 positive cells [41].

In addition, Musashi-1 is frequently expressed in both premalignant gastric lesions and invasive gastric cancer patients, *H. pylori* infection induces increased Msi-1 expression in human gastric epithelial cells [42]. Its role in tumorigenesis has been noticed in intestinal epithelial cells, yet to be tested in gastric epithelial cells. Double immunostaining experiment reveals that Musashi-1 coexpresses with stem cell markers Lgr5 cell in both normal and gastric cancer patient tissues [25], implicating it has a role in regulating normal epithelial cell differentiation, and possibly carcinogenic processes. Future studies are required to establish its possible role as a stem cell marker.

#### Smad2/3 phosphorylated at specific linker threonine (pSmad2/3L-Thr)

Recently, Smad2/3 phosphorylated at specific linker threonine residues (pSmad2/3L-Thr) has been reported as a marker for gastric stem cells [43]. pSmad2/3L-Thr immunostaining positive cells can be specifically detected in the isthmus region of both gastric corpus and antral mucosa of C57BL/6 mice. Morphologically, these cells appear undifferentiated and exhibit a high nucleus-to-cytoplasm ratio as well as much diffuse chromatin in the nuclei. Double immunostaining staining indicates that pSmad2/3L-Thr does not colocalize with cell proliferation marker Ki67, but colocalizes with CK8, indicating epithelial cell origin. pSmad2/3L-Thr immunostaining-positive cells colocalize with or near the DCAMKL1 immunostaining-positive cells which is a marker for tuft cells in gastric corpus mucosa [44], but more works are required to establish its real identity.

Putative stem cell markers, such as doublecortin and CaM kinase-like-1 (DCAMKL-1, Dclk1) is previously identified as intestinal stem cell marker, but later confirmed as a marker for gastric tuft cells [44]. Others putative markers such as identified by FACS side population with Hoechst dye staining and transcriptional analyses, including FZD7, HEY1, SMO, and ADAM17 require further investigation to confirm their real identity [45]. Future investigation to discover reliable stem cell markers are essential to understand their biology and pathobiology during *H. pylori* infection or inflammation induced cancer.

## Current understanding on *H. pylori* interacts with gastric stem or progenitor cells

**Figure 1:**
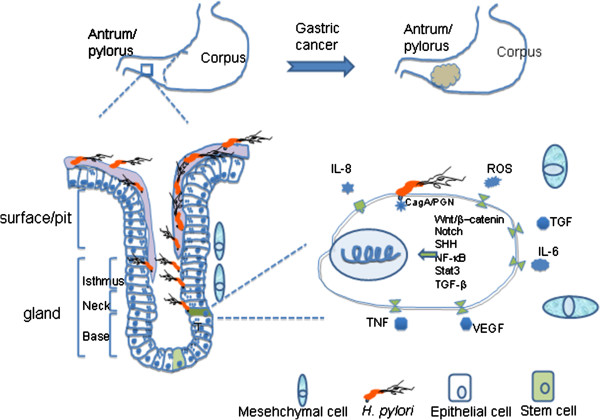
***Helicobacter pylori *infection, stem cells and gastric cancer.***H. pylori* infection induces chronic inflammation within local gastric mucosa; the proinflammatory microenvironment may disrupt stem/progenitor cell proliferation and differentiation. *H. pylori-* or inflammation-induced molecular damages in these cells could be carried to the descendants and facilitate tumor initiation. In addition, *H. pylori* CagA-, *cag*PAI-positive strains induce multiple oncogenic signaling and epigenetic alteration in epithelial cells. Persistent activation of these oncogenic pathways, alterations of epigenetic profile and deregulation of stem cell differentiation provide important molecular mechanisms toward *H. pylori*- or inflammation-induced carcinogenesis. (Image is for illustration purpose, does not scale to real size or proportion of cells, and is made by Microsoft PowerPoint software; CagA: Cytotoxin-associated antigen A; PGN: Peptidoglycan)

The stem cell hypothesis of cancer formation is that stem or progenitor cells accumulate enough molecular lesions to evade homeostatic control and result in cancer. The damages that originate from genetic and/or epigenetic processes are progressively accumulated during aging and directly contribute to cell transformation [46]. In fact, relatively little information is available about the effects of *H. pylori* infection impact on stem cells at present, although *H. pylori* has been shown to invade epithelial cell and their progenitors [47]. During chronic infection, *H. pylori* may directly or indirectly interact with gastric stem/progenitor cells, possibly disrupts their differentiation and introduces the molecular damage in these cells. Figure 1 illustrates the possible relationship of *H. pylori* infection and the initiation of gastric cancer.

Early studies was performed before the publication of stem cell markers Lgr5, Villin-promoter; in *H. pylori* infected gnotobiotic mouse model of chronic atrophic gastritis, loss of parietal cells induces amplification of multi- and oligo-potential gastric stem cells that express sialylated glycan receptors, which are recognized by *H. pylori* adhesins, and that *H. pylori* interacts with and resides within a subset of these progenitor cells [48]. GeneChip-based comparisons of the transcriptome of mouse gastric progenitor cells (GEP) infected with *H. pylori* reveal that *H. pylori* cancer-associated strain regulates the expression of GEP-associated signaling and metabolic pathways; as well as tumor suppressor genes that are associated with the development of gastric cancer in humans [49]. These results indicated potential interaction of *H. pylori* with gastric progenitor cells, although it is not clear if the interaction might lead to tumorigenesis, further investigations are required to understand the consequences and molecular mechanisms.

*H. felis* infection in mice induced gastric cancers can originate from bone marrow-derived cells that repopulate the gastric mucosa [3]. These results have recently been recapitulated by *H. pylori* infection in mice model, suggested an important source of gastric cancer [50]. The infected mice go through gastric chronic inflammation, intestinal metaplasia and develop stomach cancer. The fact that cancer formation does not require *H. pylori* infection indicate that the general inflammatory status, or microenvironment, not necessarily bacterial factors or components, may play a more important role in regulating stem cell differentiation and the initiation of gastric cancer; while *H. pylori* or *H. felis* components might promote this process. These finding are also supported by recent finding from *H. pylori* infected gerbil model, in which *H. pylori* infection induces remarkable inflammation and resulted gastric epithelial cell DNA methylation and stomach malignancy; which is caused by inflammation but not by *H. pylori* itself [51]. It is clear that epigenetic deregulation is closely related to inflammation, stem cell regulation and carcinogenesis. Future studies are sorely required to clarify these links especially during *H. pylori* infection model.

## *H. pylori* infection and changes in stem cell microenvironment or niche

The stem cell niche or microenvironment is composed of different population of cells, including not only stem cells, but also differentiated cells, soluble factors, extracellular matrix; all of them are critical for stem cell fate and differentiation [52]. Important signaling pathways such as Wnt, Notch, Hedgehog, PI3K, NF-κB, EGF, TGF-β and STAT3 have been shown to regulate stem cell renewal and maintenance, their effects overlap in both normal and cancer stem cells [53], interactions of stem cell with its surroundings are currently under intensive investigation.

*H. pylori* infection induces chronic inflammation accompanied with increased infiltration of immune cells, such as T, B, macrophage, neutrophils in the gastric mucosa, and overexpression of inflammatory mediators such as TNF-α, IL-1β, IL-6, IL-8, COX2, as well as activation of oncogenic pathways in gastric epithelial cells [2,9]. These inflammatory mediators and oncogenic pathways also regulate stem cell differentiation either directly or indirectly and are frequently deregulated in tumors [54-56]. Given the fact that gastric stem cells are such a rare population of cells and can be affected by so many intrinsic and extrinsic factors, it is obviously very complicated to identify the specific role of a signal factor played in regulating their differentiation and migration. It has been noted that NF-κB, IL-6, VEGF, HIF-1α, angiogenesis, reactive oxygen species and tissue factors all involve in the maintenance of stem cell and CSC [53] and *H. pylori* infection alters most of their expression; this provide possibilities that *H. pylori* will impact on local microenvironment and affect the stem/progenitor cell differentiation, as well as causing genetic or epigenetic damages in these cell that lead to carcinogenesis. However, experiments addressing these pathway/mediators on the gastric stem/progenitors during infection are waiting for further investigation.

Chronic inflammation also helps to recruit bone marrow derived cells (BMDCs) to the inflammatory site. BMDCs are a heterogeneous population of cells that include hematopoietic stem cells and mesenchymal stem cells (MSCs); they possess high plasticity and can differentiate into cells of diverse lineages, such as stromal myofibroblasts, epithelial cells, and can give rise to gastric epithelial cells when injected into the adult mouse stomach or early blastocysts [57]. BMDCs represent an alternative source of tumor-initiating cells from which a fraction of tumor cells possesses the capacity for self-renewal. MSCs have also been suggested to give rise to myofibroblasts or cancer-associated fibroblasts that promote tumor growth [58]. Infection with different strains of *H. pylori* is recently shown to induce MSCs migration through TNF/NF-κB-dependent pathways in epithelial cells *in vitro*, therefore provide important link for the pathogenesis [59].

It is clear that chronic inflammation and proinflammatory status are prerequisite for the initiation of malignant transformation and tumor progression in inflammatory diseases such as hepatitis, colitis, pancreatitis and gastric inflammation [52,60]. In addition, tumor mass progression involves growth and expansion of tumor cells, stromal cells, blood vessels and infiltrating inflammatory cells; their interaction propagate tumor progression. The procarcinogenic inflammatory microenvironment is critical for this process. Persistent activation of oncogenic pathways such as MAPK, Wnt, Stat3, HIF, NF-κB and epigenetic changes are hallmark for the inflammation-induced cancer.

Our understanding has expanded regarding *H. pylori* infection and gastric stem cell regulation, due to the recent investigations on these processes within the particular stem cell microenvironment. However, future studies are required to define the role of *H. pylori* or its components in disrupting stem cell niche, their self-renewal, differentiation, and finally tumorigenesis.

## Conclusion and future directions

Despite of intensive investigation, the mechanisms of *H. pylori*-induced gastric cancer remain poorly understood, the stem cell hypothesis hold the promise to elucidate the origin/initiation of gastric cancer. Study of the interactions of *H. pylori* or its virulence factors with stem/progenitor cells or BMDCs are critical steps leading toward this purpose. Furthermore, important questions such as (a) variations in disease susceptibility in different populations, (b) gastric cancer progression in relation to *H. pylori* virulence genes polymorphism, and (c) the correlation of stem cell with different types of gastric cancer are all waiting for further clarification.

*H. pylori* infection triggers inflammation, interactions of bacteria with host cell in local microenvironment also affect gastric stem/progenitor cells and their differentiation, this may potentiate oncogenic transformation. Therefore, focus on *H. pylori*-induced molecular pathogenesis and the impact of microenvironment in gastric stem or progenitor cells will be crucial to identify the molecular targets in tumor initiation and the origin of gastric cancer; investigation of which will also provide insights to uncover the carcinogenic mechanisms and options for cancer intervention and prevention.

## Competing interests

The authors declare that they have no competing interests.

## Authors' contributions

SZD and PYZ wrote and revised the manuscript; both authors read and approved the final manuscript.
